# Wnt/*β*-Catenin, Carbohydrate Metabolism, and PI3K-Akt Signaling Pathway-Related Genes as Potential Cancer Predictors

**DOI:** 10.1155/2019/9724589

**Published:** 2019-10-20

**Authors:** Pengliang Chen, Pengwei Shi, Gang Du, Zhen Zhang, Liang Liu

**Affiliations:** ^1^Department of Urology, Nanfang Hospital, Southern Medical University, Guangzhou, Guangdong 510515, China; ^2^Department of Emergency, Nanfang Hospital, Southern Medical University, Guangzhou, Guangdong 510515, China; ^3^Department of Bioinformatics, Guangzhou GenCoding Lab, Guangzhou, Guangdong 510670, China; ^4^Department of Cardiac Surgery, Guangdong Cardiovascular Institute, Guangdong General Hospital, Guangdong Academy of Medical Science, Guangzhou, Guangdong 510100, China; ^5^Department of Burns, Nanfang Hospital, Southern Medical University, Guangzhou, Guangdong 510515, China

## Abstract

Predicting the outcome after a cancer diagnosis is critical. Advances in high-throughput sequencing technologies provide physicians with vast amounts of data, yet prognostication remains challenging because the data are greatly dimensional and complex. We evaluated Wnt/*β*-catenin, carbohydrate metabolism, and PI3K-Akt signaling pathway-related genes as predictive features for classifying tumors and normal samples. Using differentially expressed genes as controls, these pathway-related genes were assessed for accuracy using support-vector machines and three other recommended machine learning models, namely, the random forest, decision tree, and k-nearest neighbor algorithms. The first two outperformed the others. All candidate pathway-related genes yielded areas under the curve exceeding 95.00% for cancer outcomes, and they were most accurate in predicting colorectal cancer. These results suggest that these pathway-related genes are useful and accurate biomarkers for understanding the mechanisms behind cancer development.

## 1. Introduction

Cancer, associated with high mortality, is indeed a serious threat to public health. One cause for the high mortality rate is nonspecific symptoms in the early stages, resulting in a poor prognosis and a high fatality rate. Thus, accurately predicting cancer is a most critical and urgent task for physicians. Because cancer is fundamentally caused by gene malfunction, utilizing their expression levels as relatively direct methods of diagnoses has attracted a great deal of research attention. To date, analyses of gene expression level data have greatly benefited cancer diagnoses and treatments [1–3]. However, the high dimensionality and noise associated with the data can make these analyses and applications challenging. To reduce these challenges, data are initially processed to identify a small subset of genes primarily responsible for the disease [4, 5]. Feature selection is reportedly a very effective method for reducing the high dimensionality of gene expression datasets [6].

Cancer biology research is rapidly finding the recurring roles of a small set of signaling cascades: the Wnt cascade, metabolism, PI3K/AKT signaling pathway, and so on. The Wnt signaling pathway is prevalent in carcinogenesis, playing an essential role in the development of various tumors [7, 8]. Indeed, current evidence suggests that up to 80% of colorectal cancers are driven by an activating mutation in the Wnt cascade [9]. Altered energy metabolism is believed to be a hallmark characteristic of cancer [10, 11]. Even in the presence of oxygen, cancer cells can reprogram their glucose metabolisms to produce energy, thus largely limiting energy metabolism to glycolysis [12]. In addition, glycolysis provides cancer cells with various metabolic precursors that promote the synthesis of amino acids, nucleotides, and lipids, leading to cancer development. The PI3K-Akt signaling pathway is most frequently activated in a variety of cancer lineages [13–15]. A range of malignancies, including ovarian, breast, colorectal, and endometrial cancers, frequently exhibit activation of the PI3K pathway through various mechanisms, including genomic mutations or alterations involving PIK3CA, PIK3R1, PTEN, AKT, TSC1, TSC2, LKB1 (also known as STK11), MTOR, and other oncogenes or tumor suppressor genes [16, 17]. This regulates key biological processes, including proliferation, the cell cycle, motility, metabolism, and genomic instability, all of which support the survival, expansion, and dissemination of cancer [18].

In conjunction with the rapidly increasing amount of gene expression data, state-of-the-art data analysis tools are being developed. Of them, machine learning (ML) methods such as random forest (RF), support-vector machine (SVM), decision tree (DT), and k-nearest neighbor (KNN) have been successfully applied to various areas of genomics research [19, 20]. Included are the expression profiles of genes [21], predicting the functional activity of genomic sequences [22], and predicting the intrinsic molecular subtypes of breast cancer [23]. Notably, RF uses highly dimensional data and data that are unbalanced and missing values [24]. An SVM is an ML algorithm that separates entities into appropriate classes using a hyperplane [25]. In cancer research, it has been used successfully to classify people as those with and without cancer based on microarray expression data [26].

These methods were used in this study to predict the cancer state from gene expression data from various types of cancer. Given the significant roles of these cancers, pathway-related genes were used as alternative features.

## 2. Materials and Methods

### 2.1. Data Acquisition

Genetic data were downloaded from The Cancer Genome Atlas, a publicly accessible dataset (https://cancergenome.nih.gov/). The microarray expression data included colorectal cancer (1222 samples, 1109 tumorous), gastric cancer (407 samples, 375 tumorous), and breast cancer (440 samples, 410 tumorous). Detailed information about the data is shown in Table 1, and the number of pathway-related genes in the candidate cancers is shown in Table 2.

### 2.2. Data Preprocessing

Data preprocessing is a crucial step in ML, and errors at this stage can lead to misleading prediction results. This study included the following preprocessing steps: Data were normalized for each sample by first transforming the data using the log ratio base 2 and then, for each probe, calculating the median of the log-summarized values from all samples and subtracting it from each sample. Missing values were replaced with the attribute mean.

### 2.3. Feature Selection

For clinical use, the number of cancer samples was unbalanced in comparison with the number of features, possibly leading to a high risk of overfitting and degrading the classification performance, thus significantly affecting predication accuracy. However, effective feature selection is a method used to address this challenge [27]. Considering the importance of pathways in tumorigenesis, three pathway-related genes were selected as candidate features. They were the Wnt/*β*-catenin, carbohydrate metabolism, and PI3K-Akt signaling pathways. Simultaneously, significantly differentially expressed genes (DEGs) were used as controls for comparing the features used for cancer classification. These DEGs have been previously employed in cancer prediction studies, and the findings support their use as valid features. The DESeq *R* package [28] was used to identify DEGs. Our criteria were a *P* value of less than 0.001 and a log 2 fold change of 4 or more. Notably, the pathway-related genes were derived from the Kyoto Encyclopedia of Genes and Genomes (http://www.kegg.jp/) analysis.

### 2.4. Conventional Machine Learning Algorithms

All four widely used classification methods (SVM, RF, DT, and KNN) were adopted. In the SVM method, the parameter *C* was assigned a value of either 0.1, 1, 10, or 100, and the kernel function was either “linear,” “rbf,” “poly,” or “sigmoid.”

In the KNN method, the number of neighbors was assigned as 3, 5, or 7, and the Euclidean distance, Manhattan distance, and Minkowski distance were combined to train the model.

In the DT algorithm, CART was used, and the maximum tree depth was 5 or 10. In the RF model, the numbers of DTs were 5, 10, or 50 and the numbers of features were 2, 4, 10, or 20.

## 3. Results

### 3.1. General Classification Workflow

**Figure 1:**
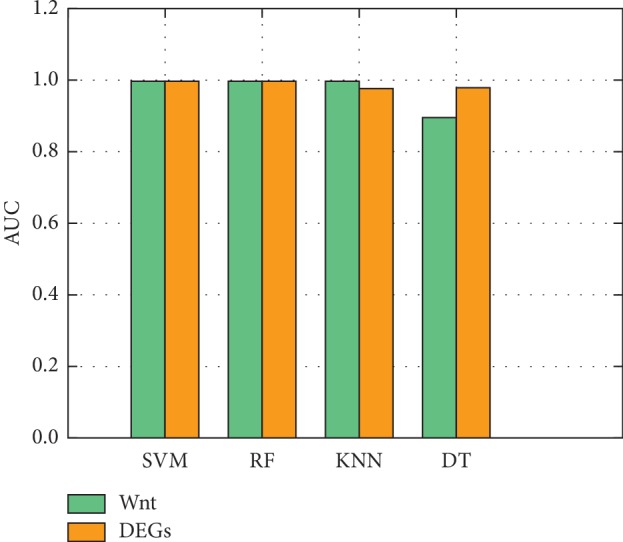
Average areas under the curve (AUCs) for Wnt signal pathway-related genes and differentially expressed genes (DEGs) using four machine learning algorithms to predict colorectal cancer from gene expression data. For the pathway genes, support-vector machine (SVM) yields an AUC of 99.49%, decision tree (DT) yields 89.45%, random forest (RF) yields 99.49%, and k-nearest neighbor (KNN) yields 99.42%. For DEGs, SVM yields 99.49%, DT, 99.49%, RF, 96.18%, and KNN, 97.85%.

Data were extracted from the Kyoto Encyclopedia of Genes and Genomes database. Specifically, 142, 356, and 350 elements (pathway-related genes) were found for the Wnt, carbohydrate metabolism, and PI3K-Akt signaling pathways, respectively. In addition, 314, 241, and 133 DEG parameters were included for colorectal, breast, and gastric cancer, respectively. To evaluate the cancer predictive ability of these pathway-related genes, the workflow shown in Figure 1 was implemented. Before training the model, all data were subjected to pretraining the model using an autoencoder without labels. This step was designed to improve model performance, avoid random initialization of the weights, and select the candidate model architecture associated with the minimum mean square error.

### 3.2. Wnt Pathway-Related Genes Score as High as DEGs in Predicting Colorectal Cancer

**Figure 2:**
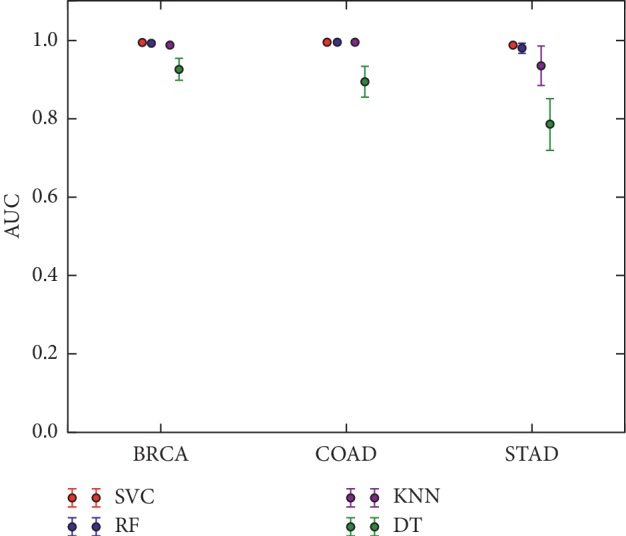
Performance of the Wnt signal pathway-related genes in three types of cancers—colorectal cancer, breast cancer, and gastric cancer—using four machine learning algorithms.

Detailed information about the relative sample and pathway-related genes is shown in Tables 1 and 2. The prediction performances of the entire set of Wnt pathway-related genes and of the DEGs were evaluated using three common metrics: precision, recall, and accuracy. Results are shown in Tables 3 and 4. Scores using Wnt pathway-related genes are comparable to those found using DEGs, achieving approximately 95% accuracy for classifying colorectal cancer regardless of the ML method used (Figure 2).

### 3.3. Wnt Pathway-Related Genes Are Efficient Predictors of Cancer

**Figure 3:**
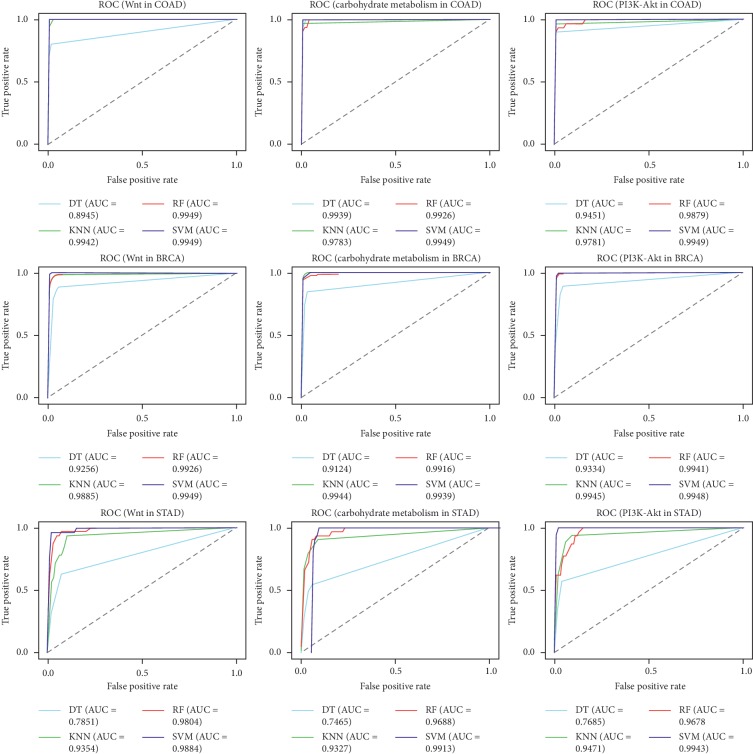
Receiver operating characteristic curves for the Wnt signaling pathway-, PI3K-Akt signaling pathway-, and carbohydrate metabolism signal pathway-related genes for the three datasets.

Based on these results, we hypothesized that the Wnt pathway is potentially a feature that can be adopted for cancer detection. To test this, it was evaluated with common cancers such as breast and gastric cancers. Similar procedures and algorithms were selected, and DEGs were used as controls. Not surprisingly, results using the Wnt pathway-related genes were similar to those using the control group: the area under the curve (AUC) exceeded 94.00%. It is worth noting that Wnt pathway-related genes in breast cancer outperformed those in gastric cancer (AUC values of approximately 98% and 95%, respectively Figure 3).

### 3.4. Carbohydrate Metabolism and PI3K-Akt Signaling Pathways Can Predict Cancer Status

**Figure 4:**
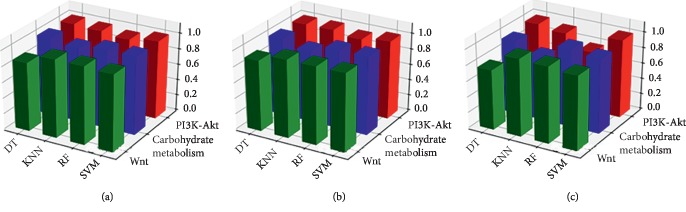
3D bar plots of the three candidate features in various types of cancers. The *z*-axis indicates percent area under the curve. (a) COAD, (b) BRCA, and (c) STAD.

It is unknown whether other cancer-related pathways can predict cancer status. Thus, a set of carbohydrate metabolism and PI3K-Akt signaling pathway-related genes were chosen to test their abilities to predict our candidate cancers. The carbohydrate metabolism pathway-related genes scored highest for colorectal cancer followed by breast cancer and gastric cancer. Similar results were found using ML methods: AUC values were 98.28%, 97.30%, 96.07%, and 96.31% when using SVM, RF, DT, and KNN, respectively. Interestingly, the PI3K-Akt signaling pathway-related genes performed similarly. Both carbohydrate metabolism and PI3K-Akt signaling pathways yielded AUCs above 96.00%, implying that both pathways can detect cancer with great accuracy (Table 5). Of note, the SVM and RF methods outperformed DT and KNN in cancer detection (Figure 4). Taken together, these results indicate that these three pathway-related genes can be vital features for cancer prediction and that these pathways vary in predictive power. We believe that most pathway-related genes are promising features that could be used for early cancer diagnoses.

## 4. Discussion

Increasing evidence indicates that colorectal cancer is often initiated by an activating mutation in the Wnt cascade. The correlation between the Wnt pathway and colorectal cancer prompted our investigation into whether Wnt pathway-related genes serve as features for detecting colorectal cancer. Thus, we designed this study to take advantage of various conventional ML models and cancer-related pathways for predicting cancer. Results show that these three pathway-related genes could be used as features for cancer prediction; they yielded results equal to those of DEGs.

Given the complexity and high mortality of cancer, the accurate early diagnosis of a cancer type can facilitate clinical management. Only relatively recently has cancer researchers attempted to apply ML for cancer prediction and prognosis [29–31]. Most previous work employed ML methods for modeling cancer progression and then identified informative factors used in a classification scheme and attempted to develop a set of classifiers for feature selection. Conventional ML algorithms require engineering domain knowledge to identify features from raw data, whereas ML automatically extracts simple features from the input data using an all-purpose learning procedure. These simple features are mapped into outputs using a complex architecture composed of a series of nonlinear functions (i.e., “hierarchical representations”) to maximize the predictive accuracy of the model. This measure can be improved using rich information contained in the biological research. We aimed to fill this void by assessing pathway-related genes for their performances in cancer prediction and identification.

We demonstrated that three cancer-related pathways (the Wnt signaling pathway, carbohydrate metabolism signaling pathway, and PI3K-Akt signaling pathway) have high predictive accuracy compared with DEGs for cancer prediction and identification. Furthermore, their performances were similar regardless the ML algorithm used. The use of DEGs as features has been previously documented. However, the outcomes suggest that all three pathway-related genes can be used as features for cancer detection. By assessing various cancer types, we observed that the features perform best for colorectal cancer followed closely by breast cancer and then gastric cancer. We speculated that the function of pathway-related genes in various cancer types can vary and are more serious in colorectal cancer. Results also show that these three pathway-related genes achieved different performances for one cancer type, and this can result in contributions of their compositions that vary based on the type of tumorigenesis.

Finally, these results demonstrate that the SVM and RF algorithms are superior to those of DT and KNN in genomics research. This variation might be because the classifier differs from one problem to another (e.g., the SVM model tends to meet rule-matching well when hundreds of thousands of dimensions exist, as in this study, whereas DT and KNN depend largely on feature selection in nonlinearly related variables). Unlike studies using other ML methodologies, this study offers additional insights on feature extraction for cancer classification. Each of the novel observations we found are worthy of further investigation.

## 5. Conclusions

We propose that pathway-related genes have the potential to be used as biomarkers for cancer prediction. We demonstrated that the Wnt signaling pathway, carbohydrate metabolism signaling pathway, and PI3K-Akt signaling pathway can be incorporated into ML models to achieve better prediction performance. The proposed features have the potential to facilitate preoperative care of patients with cancer.

## Figures and Tables

**Table 1:** Clinical features of patients in The Cancer Genome Atlas (TCGA) dataset.

Clinical factor	TCGA			
	COAD	BRCA	STAD	PRAD
	*n* = 440	*n* = 1222	*n* = 407	*n* = 550
Patient count (selected/original)	387/410	1089/1109	375/375	493/498
Age (years, mean ± SD)	65.73 ± 13.06	58.46 ± 13.20	65.83 ± 10.65	65.83 ± 10.65
Sex (male/female/−)	201/186	12/1077	241/134	493/0
Death (dead/alive/−)	82/304/1	152/937	150/225	10/483
Overall survival (months, mean ± SD)	28.46 ± 26.27	40.96 ± 30.17	19.32 ± 18.08	35.76 ± 25.89

*Note.* Selected patients included those with clinical characteristics after removing normal, replicate, and missing features from the total sample used in the model.

**Table 2:** Elements of pathway-related genes in candidate cancers.

Datasets	Typical			Nontypical			DEGs
	Wnt	Ca-Me	PI3K	TLR	TH	RPSC	
COAD (total/selected)	143/142	356/356	351/350	104/102	116/116	139/139	314/314
BRCA (total/selected)	143/142	356/356	351/350	104/102	116/116	139/139	241/241
STAD (total/selected)	143/142	356/356	351/350	104/102	116/116	139/139	133/133
PRAD (total/selected)	143/142	356/356	351/350	104/102	116/116	139/139	169/169

*Note*. COAD, colorectal cancer; BRCA, breast cancer; STAD, gastric cancer; PRAD, prostate cancer; Wnt, Wnt signaling pathway; Ca-Me, carbohydrate metabolism signaling pathway; PI3K, PI3K-Akt signaling pathway; TLR, toll-like receptor signaling pathway; TH, thyroid hormone signaling pathway; RPSC, signaling pathways regulating pluripotency of stem cells; DEGs, differentially expressed genes.

**Table 3:** Performances of pathway-related genes and DEGs in training set.

Genes	Training set															
	COAD (%) (normal/tumor = 21/287)				BRCA (%) (normal/tumor = 78/777)				STAD (%) (normal/tumor = 21/263)				PRAD (%) (normal/tumor = 36/349)			
	SVM	RF	DT	KNN	SVM	RF	DT	KNN	SVM	RF	DT	KNN	SVM	RF	DT	KNN
Wnt	100.00	99.73	91.97	99.82	99.85	99.16	86.78	99.50	96.30	99.14	79.55	93.76	94.97	90.73	69.88	89.58
Ca-Me	100.00	99.82	97.15	100.00	99.78	98.10	93.21	99.10	99.42	95.30	74.56	95.31	97.15	91.89	77.56	89.18
PI3K	100.00	100.00	96.80	100.00	99.88	99.04	89.92	99.64	99.42	98.19	81.96	97.91	95.07	94.81	71.84	90.97
TLR	100.00	99.27	84.45	100.00	99.63	98.93	86.50	98.59	96.95	91.94	81.39	95.37	94.08	89.10	65.20	86.79
TH	100.00	99.91	90.54	100.00	99.81	98.21	88.77	98.78	99.23	94.79	86.93	97.51	94.85	92.20	69.92	90.62
RPSC	100.00	99.93	97.15	99.64	99.84	99.22	90.48	99.01	98.41	99.23	81.48	97.20	93.63	94.78	78.34	89.11
DEGs	100.00	99.96	96.95	100.00	99.85	99.89	96.94	98.68	99.32	99.66	88.81	97.61	96.48	95.53	83.60	94.33

**Table 4:** Performances of pathway-related genes and DEGs in test sets.

Genes	Test set															
	COAD (%) (normal/tumor = 9/123)				BRCA (%) (normal/tumor = 35/332)				STAD (%) (normal/tumor = 11/112)				PRAD (%) (normal/tumor = 16/149)			
	SVM	RF	DT	KNN	SVM	RF	DT	KNN	SVM	RF	DT	KNN	SVM	RF	DT	KNN
Wnt	100.00	99.90	94.03	99.86	99.95	97.64	88.79	100.00	99.26	98.70	79.13	99.75	95.76	95.55	76.65	95.26
Ca-Me	100.00	100.00	99.18	100.00	99.96	99.50	88.42	100.00	99.10	97.88	75.04	97.44	94.75	96.95	79.67	95.11
PI3K	100.00	100.00	100.00	99.95	99.92	99.43	98.41	99.66	99.35	98.53	84.57	97.44	96.56	91.61	86.01	95.91
TLR	100.00	97.01	88.48	100.00	99.87	99.35	90.17	99.44	98.62	98.01	86.36	95.37	93.20	92.05	78.56	86.79
TH	100.00	99.72	99.18	99.86	99.93	98.86	83.38	99.57	99.43	98.45	80.92	97.93	96.60	89.53	64.91	91.42
RPSC	100.00	99.86	94.03	99.95	99.93	99.93	94.13	99.84	99.26	98.25	83.68	98.74	94.67	93.12	79.88	95.05
DEGs	100.00	100.00	100.00	100.00	100.00	99.96	99.03	99.29	99.35	99.10	88.67	96.02	93.37	92.76	85.48	91.23

*Note*. COAD, colorectal cancer; BRCA, breast cancer; STAD, gastric cancer; PRAD, prostate cancer; Wnt, Wnt signaling pathway; Ca-Me, carbohydrate metabolism signaling pathway; PI3K, PI3K-Akt signaling pathway; TLR, toll-like receptor signaling pathway; TH, thyroid hormone signaling pathway; RPSC, signaling pathways regulating pluripotency of stem cells.

**Table 5:** Performance of candidate pathway-related genes in cancer prediction.

Classifiers	Pathway-related genes								
	Wnt			Carbohydrate metabolism			PI3K-Akt		
	COAD (%)	BRCA (%)	STAD (%)	COAD (%)	BRCA (%)	STAD (%)	COAD (%)	BRCA (%)	STAD (%)
SVM	99.49	99.49	98.84	99.49	99.39	99.13	99.49	99.48	99.43
RF	99.49	99.26	98.04	99.26	99.16	96.88	98.79	99.41	96.78
DT	89.45	92.56	78.51	99.39	91.24	74.65	94.51	93.34	76.85
KNN	99.42	98.85	93.54	97.83	99.44	93.27	97.81	99.45	94.71

*Note*. Tumor samples in the positive group versus the normal samples. COAD, colorectal cancer; BRCA, breast cancer; STAD, gastric cancer.

## Data Availability

Genetic data were downloaded from The Cancer Genome Atlas, a publicly accessible dataset (https://cancergenome.nih.gov/), and the pathway-related genes were derived from the Kyoto Encyclopedia of Genes and Genomes (http://www.kegg.jp/) analysis.
